# PRDX6 Drives Breast Cancer Progression Through Mitochondrial Biosynthesis and Oxidative Phosphorylation

**DOI:** 10.1002/cam4.71005

**Published:** 2025-06-30

**Authors:** Mei Dai, Danhua Zhang

**Affiliations:** ^1^ Department of General Surgery The Second Xiangya Hospital, Central South University Changsha Hunan China; ^2^ Clinical Research Center for Breast Disease of Hunan Province Changsha Hunan China

**Keywords:** breast cancer, mitochondria, oxidative phosphorylation (OXPHOS), PRDX6, tumorigenesis

## Abstract

**Background:**

Peroxiredoxin 6 (PRDX6) scavenges reactive oxygen species (ROS) and plays a key role in antioxidant defense. Although PRDX6 is involved in various cancers, its role in breast cancer (BRCA) remains unclear.

**Methods:**

Cell proliferation was assessed using CCK‐8, EdU staining, and colony formation assays. Migration and invasion were evaluated via wound‐healing and transwell assays. ROS levels and mitochondrial membrane potential were measured by fluorescence microscopy or flow cytometry. Oxidative phosphorylation (OXPHOS) activity was determined by ATP production and NAD^+^/NADH ratio. Mitochondria were visualized by TEM, and mitochondrial complex subunits were detected by quantitative real‐time PCR and Western blotting. In vivo effects were evaluated using a xenograft tumor model.

**Results:**

Although PRDX6 was downregulated in BRCA overall, it showed elevated expression in aggressive subtypes and advanced‐stage tumors, correlating with poor prognosis. Overexpression of PRDX6 enhanced BRCA cell proliferation, migration, and invasion. PRDX6 reduced ROS levels, upregulated mitochondrial transcription factor A (TFAM) expression, and promoted mitochondrial complex subunit expression and OXPHOS. Inhibition of TFAM led to a decrease in the expression of some of the mitochondrial complex subunits, which reversed the pro‐carcinogenic phenotype of the tumor. PRDX6 also promoted tumor growth in vivo.

**Conclusion:**

PRDX6 maintains intracellular homeostasis by reducing ROS and promotes mitochondrial biogenesis and OXPHOS through TFAM‐dependent and ‐independent pathways, driving BRCA progression.

## 1 Introduction

Breast cancer (BRCA) is the predominant malignant neoplasm in the female population and a principal etiology of cancer‐related fatalities [1]. BRCA is heterogeneous and can be classified into three distinct molecular subtypes based on the expression of estrogen receptor (ER), progesterone receptor (PR), and human epidermal growth factor receptor 2 (HER2): ER‐positive, HER2‐positive, and triple‐negative breast cancers (TNBCs) [2]. Despite the evolution of therapeutic modalities for BRCA, the repertoire of molecular biomarkers for early diagnosis, prognostic stratification, and predictive response to treatment is constrained. This scarcity of biomarkers results in a significant number of patients enduring the sequelae of postoperative relapse, metastatic spread, and the emergence of chemoresistance [3, 4]. Therefore, elucidating the intricate molecular pathways involved in the pathogenesis and progression of BRCA is imperative for the amelioration of patient prognoses.

Peroxiredoxin 6 (PRDX6) is a member of the peroxiredoxin family and plays a dual role as both a glutathione peroxidase (GPx) and a calcium‐independent phospholipase A2 (iPLA2) [5]. As a selenium‐independent peroxidase, PRDX6 can effectively neutralize reactive oxygen species (ROS) and maintain redox homeostasis, which is an integral part of the antioxidant defense mechanism [5]. Previous research has demonstrated the overexpression of PRDX6 in various malignancies, including cervical cancer [6], lung cancer [7], and colorectal cancer [8], where it contributes significantly to tumorigenesis and represents a potential target for diagnostic and therapeutic targeting.

While previous research has demonstrated that PRDX6 augments the invasiveness of BRCA cells by upregulating the expression of uPAR, Ets‐1, MMP‐9, and RhoC, and downregulating TIMP‐2, the precise underlying mechanisms have not been fully elucidated [9]. Herein, we demonstrated that PRDX6 enhanced the proliferation and invasive capacity of BRCA cells and promoted the growth of subcutaneous tumors in mice. The antioxidant function of PRXD6 reduced the level of ROS in the tumor cells, allowing it to facilitate mitochondrial biosynthesis and oxidative phosphorylation (OXPHOS) processes through both TFAM (mitochondrial transcription factor A)‐dependent and ‐independent pathways, thereby meeting the high energy demands of tumor cell growth.

## 2 Materials and Methods

### 2.1 Cell Culture, Transfection, and Infection

The human BRCA cell lines SUM159PT and MCF‐7 were purchased from ATCC. Cells were cultured in high‐glucose DMEM (Procell, Wuhan, China) supplemented with 10% fetal bovine serum (Gibco, USA) and 100 U/mL penicillin/streptomycin (Procell, Wuhan, China) in a sterile incubator at 37°C with 5% CO_2_. Full‐length PRDX6 cDNA was cloned into the pCHD lentiviral vector. PRDX6 overexpression lentiviral particles and control lentiviral particles were produced in 293 T cells and used to infect tumor cells. Stable PRDX6‐overexpressing cells were selected with 2 mg/mL puromycin for 2 weeks. Tumor cells were seeded into 6‐well plates and incubated overnight before transfection with small interfering RNA (siRNA). According to the manufacturer's protocol, siRNA and negative control (NC) were transfected into tumor cells using Lipofectamine 2000 reagent (Invitrogen, USA). The synthetic sequences for siRNA targeting PRDX6 and the control were as follows:

siPRDX6‐1 forward: 5′‐CCGAAAGGAGUCUUCACCAAATT‐3′.

reverse: 5′‐UUUGGUGAAGACUCCUUUCGGTT‐3′.

siPRDX6‐2 forward: 5′‐UGGUCCUGAUAAGAAGCUGAATT‐3′.

reverse: 5′‐UUCAGCUUCUUAUCAGGACCATT‐3′.

siPRDX6‐3 forward: 5′‐CCCAUCAUCGAUGAUAGGAAUTT‐3′.

reverse: 5′‐AUUCCUAUCAUCGAUGAUGGGTT‐3′.

siTFAM forward: 5′‐ GGACUUUCACCUACGGCAATT‐3′.

reverse: 5′‐UUGCCGUAGGUGAAAGUCCTT‐3′.

NC forward: 5′‐UUCUCCGAACGUGUCACGUTT‐3′.

reverse: 5′‐ACGUGACACGUUCGGAGAATT‐3′.

### 2.2 Quantitative Real‐Time PCR (RT‐qPCR)

Total RNA was extracted from the cells using RNAex Pro RNA Extraction Reagent (Accurate Biology, AG21101, Hunan, China) according to the manufacturer's instructions. First‐strand cDNA was synthesized using the Evo M‐MLV Reverse Transcription Kit (Accurate Biology, AG11705, Hunan, China). The first‐strand cDNA was synthesized using the Evo M‐MLV Reverse Transcription Kit (Accurate Biology, AG11705, Hunan, China), and the qPCR reaction was carried out using the SYBR Green Pro Taq HS Premixed qPCR Kit (Accurate Biology, AG11701, Hunan, China). The results were normalized to GAPDH as the reference gene and expressed as the relative fold‐change value compared with the control according to the ΔΔCT method. The qPCR primer sequences are shown in Table S1.

### 2.3 Western Blot

Total protein was extracted using RIPA buffer (New Cell & Molecular Biotech, WB3100, Jiangsu, China), and protein concentration was determined by the BCA assay (New Cell & Molecular Biotech, WB6501, Jiangsu, China). Samples were separated by 10% SDS‐PAGE and transferred onto PVDF membranes (Millipore, USA). The membranes were probed with the following antibodies: anti‐PRDX6 rabbit monoclonal antibody (1:1000, RM5184, Biodragon), anti‐TFAM recombinant rabbit monoclonal antibody (1:1000, JM32‐44, HUABIO), anti‐MTCO2 rabbit polyclonal antibody (1:1000, 55,070‐1‐AP, Proteintech), anti‐ATP5A1 rabbit polyclonal antibody (1:1000, 14,676‐1‐AP, Proteintech), anti‐NDUFB8 rabbit polyclonal antibody (1:1000, 14,794‐1‐AP, Proteintech), anti‐phospho‐AKT (Ser473) rabbit mAb (1:1000, 4060, CST), anti‐phospho‐mTOR (Ser2448) rabbit mAb (1:1000, 5536, CST), anti‐AKT1 recombinant rabbit monoclonal antibody (1:1000, ST05‐09, HUABIO), anti‐mTOR recombinant rabbit monoclonal antibody (1:1000, SU30‐00, HUABIO), anti‐GAPDH rabbit polyclonal antibody (1:1000, GB15004‐100, Servicebio) and goat anti‐rabbit recombinant secondary antibody (1:10000, AWS0002, Abiowell).

### 2.4 Cell Counting Kit‐8 Proliferation Assay

Cells were seeded at a density of 2 × 10^3^ cells per well in a 96‐well plate and incubated overnight, with five replicate wells for each group. The cell proliferation rate was assessed using the Cell Counting Kit‐8 (TargetMol, C0005, Shanghai, China). The optical density (OD) at 450 nm was measured every 24 h for a total of 4 days, after a 2‐h incubation at 37°C in a 5% CO atmosphere.

### 2.5 Colony Formation Assay

Cells were seeded at a density of 800 cells per well in 6‐well plates and cultured for 10 days. Colonies were fixed with methanol and stained with 0.1% crystal violet for 30 min. After drying the plates, the number of colonies was counted and photographed.

### 2.6 EdU Staining

Cell proliferation was assessed using the BeyoClick EdU kit (Beyotime, C0071S, Shanghai, China). Cells were treated with 10 μM EdU for 2 h, fixed with 4% PFA, and permeabilized in PBS/0.3% Triton X‐100 for 15 min. After three washes with 3% BSA buffer, click reactions were performed for 30 min in the dark. Cell nuclei were stained with Hoechst 33,342 (1:1000) for 10 min in the dark. After three washes, fluorescence was detected. The excitation/emission maximum wavelength of azide 488 is 495/519 nm, while Hoechst 33,342 emits blue fluorescence at a maximum wavelength of 346/460 nm.

### 2.7 Matrigel‐Transwell

Cells (1 × 10^4^) were seeded in serum‐free medium in the upper chamber of an 8 μm transwell (Corning, 3464, USA) coated with Matrigel (YEASEN, 40183ES08, Shanghai, China), while the lower chamber contained medium with 20% FBS. After 24 h of incubation, cells were fixed with formaldehyde. Nonadherent cells in the upper chamber were carefully wiped away, and cells attached to the membrane were stained with crystal violet and counted.

### 2.8 Wound‐Healing Assay

Cells were seeded in 6‐well plates at a density of 4 × 10^5^ cells per well and grown until confluent. After washing with phosphate‐buffered saline (PBS) to remove nonadherent cells, a sterile 20 μL pipette tip was used to create scratch wounds in each well. Images were captured at 0 h and 48 h in the same area to measure the distance of wound closure, indicating cell migration ability.

### 2.9 Intracellular ROS Production Assays

Cells were seeded in 6‐well plates at a density of 3 × 10^5^ cells per well and incubated overnight. According to the instructions, cells were incubated with serum‐free medium containing 10 μM DCFH‐DA (Beyotime, S0033S, Shanghai, China) for 30 min and then washed three times with PBS. Nuclei were stained with Hoechst 33,342 diluted 1:1000 (1000×) in PBS for 10 min at room temperature in the dark. After washing three times with PBS, the fluorescence intensity of DCF was detected at 488 nm using a fluorescence microscope or flow cytometer.

### 2.10 Mitochondrial Potential Detection

Cells were seeded in 6‐well plates at a density of 3 × 10^5^ cells per well and incubated overnight. Following the manufacturer's instructions, cells were washed once with PBS, and then 1 mL of culture medium was added, followed by 1 mL of JC‐1 (Beyotime, C2006, Shanghai, China) staining working solution. The cells were mixed and incubated in a 37°C incubator for 20 min; then the supernatant was discarded, and cells were washed twice with JC‐1 staining buffer. Finally, 1 mL of culture medium was added, and fluorescence intensity was detected using a fluorescence microscope.

### 2.11 ATP Production Measurement

ATP levels in cells are determined by the luciferin‐luciferase system using the ATP Assay Kit (Beyotime, S0026, Shanghai, China). Briefly, cell lysates are centrifuged at 12,000 g for 10 min at 4°C, and a portion of the supernatant is used to determine protein concentration by BCA. The remaining supernatant is incubated for 5 min in ATP assay buffer containing firefly luciferin and firefly luciferase reagents. Chemiluminescence detection was then performed using a multifunctional enzyme marker (Thermo Fisher Scientific, CA, USA). ATP levels were normalized to the measured protein concentration.

### 2.12 Intracellular NAD+/NADH Ratio Measurement

The intracellular NAD+/NADH ratio was measured using the NAD+/NADH Assay Kit (Beyotime, S0175, Shanghai, China) following the manufacturer's instructions. A total of 1 × 10^6^ cells were harvested and directly sorted into 200 μL of lysis buffer using NADH/NAD+ extraction buffer. The protein concentration was determined using the BCA assay. The optical density at 450 nm (OD 450 nm) was measured using a multiwell spectrophotometer. Based on the standard curve generated from the NADH standards provided in the kit, the NADH content and the total NAD+ and NADH content in each sample were calculated. The NAD+/NADH ratio was then determined, and the obtained data were normalized to the measured protein concentration.

### 2.13 Transmission Electron Microscopy

Cells were fixed overnight at 4°C in electron microscopy fixation solution (Servicebio, G1102, Wuhan, China), followed by three washes with 0.1 M phosphate buffer (PB, pH 7.4). The samples were then fixed in 1% osmium tetroxide (prepared in 0.1 M PB, pH 7.4) at room temperature in the dark for two hours. Dehydration was performed sequentially in ethanol solutions with concentrations of 30%, 50%, 70%, 80%, 95%, and 100%, followed by two 15‐min incubations in 100% acetone. The samples were infiltrated with a 1:1 mixture of acetone and 812 embedding resin (SPI, 90529–77‐4, Pennsylvania, USA) at 37°C for 2–4 h, then with a 1:2 mixture at 37°C overnight, and finally with pure 812 embedding resin at 37°C for 5–8 h. The samples were embedded in pure 812 resin within embedding molds, cured overnight at 37°C, and then polymerized in a 60°C oven for 48 h. Ultrathin sections (80 nm) were prepared using an ultramicrotome (Leica UC7, Milan, Italy) and mounted onto 150‐mesh copper grids coated with Formvar film. The sections were observed in a HITACHI TEM (HT7800, Marunouchi, Japan).

### 2.14 Tumor Xenograft Experiments

Five‐week‐old female nude mice (immunodeficient) were obtained from the Experimental Animal Centre of the Second Xiangya Hospital, Central South University. The experimental protocol was approved by the Animal Ethical and Welfare Committee of the Second Xiangya Hospital (Approval No. 20241090), and all mice were maintained in accordance with institutional ethical guidelines. SUM159PT cells (5 × 10^6^ cells in 100 μL) transfected with either the PRDX6 overexpression plasmid or an empty vector were subcutaneously injected into the lateral abdominal region of the mice. Tumor dimensions were measured using calipers every three or four days, and tumor volume was calculated using the formula: V = (length×width^2^)/2. Mice were executed by cervical dislocation.

### 2.15 Hematoxylin–Eosin (H&E) Staining

Tumor tissues were harvested and fixed in 4% paraformaldehyde for 24 h and then dehydrated in an automated tissue processor for 16 h. Following routine paraffin embedding, 5‐μm sections were cut and baked at 60°C for 1 h. Sections were deparaffinized with xylene, ethanol, and water. Hematoxylin staining was applied for 10 min, followed by rinsing to remove residual dye. Cells were differentiated with 5% acetic acid and counterstained with eosin. After drying, sections were mounted with neutral gelatin and imaged using a microscope system.

### 2.16 Immunohistochemical (IHC) Staining

Paraffin‐embedded sections (4 μm) were deparaffinized and rehydrated. Antigen retrieval was done in citrate buffer (pH 6.0) for 15 min. Endogenous peroxidase was blocked with 3% H2O2. Sections were incubated with anti‐Ki67 recombinant rabbit monoclonal antibody (1:500; ST50‐01, HUABIO) overnight at 4°C, followed by HRP‐conjugated secondary antibody for 30 min. Color development was with 3‐amino‐9‐ethylcarbazole for 15 min and hematoxylin counterstaining. Staining intensity was scored by two pathologists based on the product of positive cell percentage and staining intensity.

### 2.17 Statistical Analyses

Statistical analyses of the cell experiments were performed using GraphPad Prism 9. Data are expressed as the mean ± SD of at least three independent experiments. Statistical significance was determined using Student's t‐test (two‐tailed) and one‐way ANOVA as appropriate. A P‐value of less than 0.05 was considered statistically significant.

## 3 Results

### 3.1 PRDX6 Was Associated With Poor Prognosis in Patients With BRCA

**FIGURE 1:**
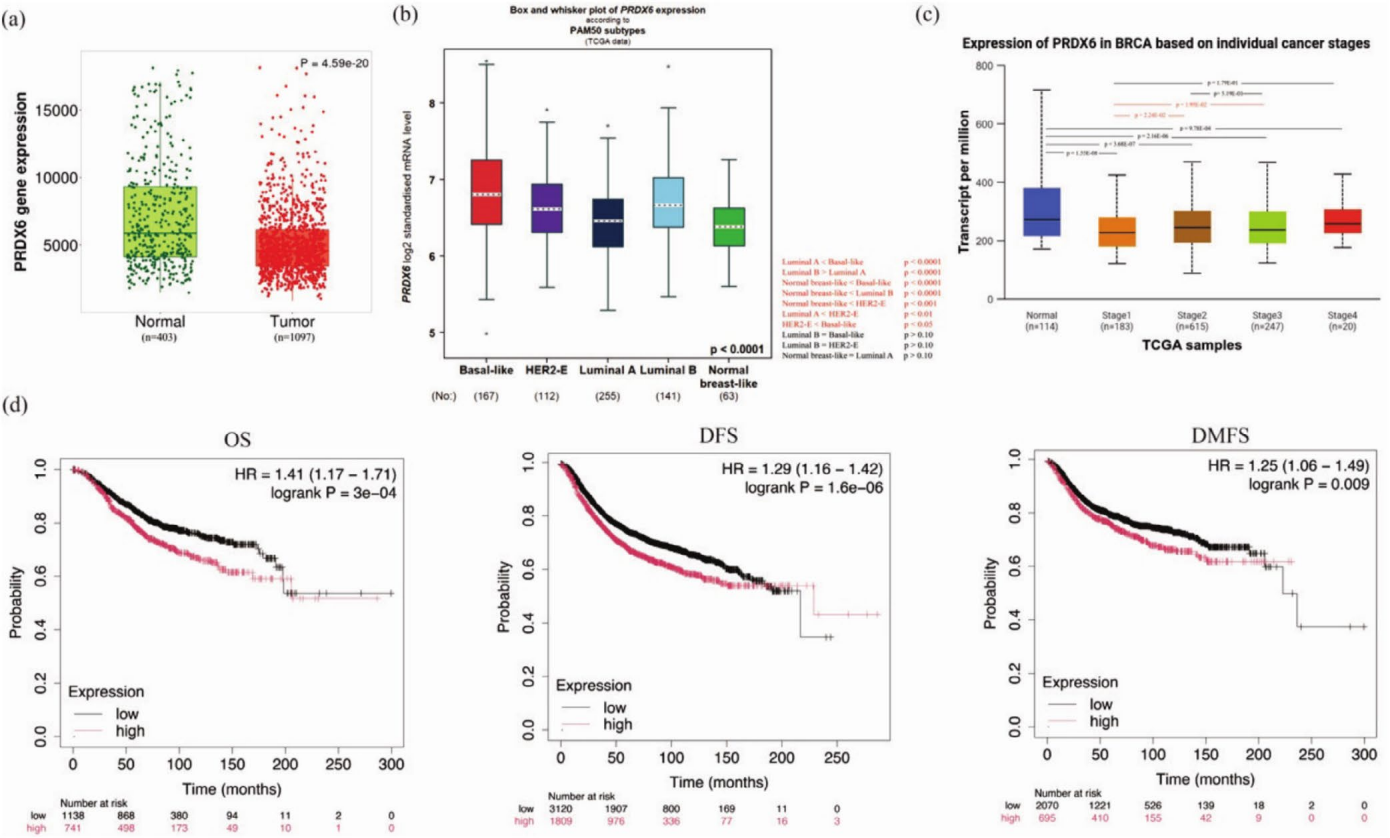
High PRDX6 expression was associated with poor prognosis in BRCA patients. (a) The mRNA expression profiles of PRDX6 in BRCA tissues compared with normal tissues. (b) PRDX6 expression according to PAM50 typing. (c) PRDX6 expression based on BRCA cancer stage. (d) The OS, DFS, and DMFS were determined according to PRDX6 expression in BRCA patients by the Kaplan–Meier database. BRCA, breast cancer; OS, overall survival; DFS, disease‐free survival; DMFS, distant metastasis‐free survival.

To evaluate the clinical significance of PRDX6 in BRCA, we first analyzed its mRNA expression levels in BRCA tissues and normal breast tissues using the TNMplot database [10], based on transcriptomic data from the Genomic Data Commons and Genotype‐Tissue Expression projects. The results revealed that PRDX6 expression was significantly higher in normal tissues than in tumor tissues (*p* = 4.59e‐20; Figure 1a). Subtype‐specific analysis based on PAM50 classification showed that PRDX6 expression was highest in Basal‐like and Luminal B subtypes, and lowest in the Luminal A subtype (Figure 1b). Furthermore, PRDX6 expression was up‐regulated in highly staged tumors: patients with Stage2 and Stage3 had higher PRDX6 expression than patients with Stage1 (Figure 1c).

Further analysis using the Kaplan–Meier Plotter database [11] demonstrated that high PRDX6 expression was significantly associated with worse overall survival (OS, *p* = 3e‐04), disease‐free survival (DFS, *p* = 1.6e‐06), and distant metastasis‐free survival (DMFS, *p* = 0.009; Figure 1d) in BRCA patients. OS analyses based on RNA‐seq were consistent (Figure S1). Collectively, these findings suggest that elevated PRDX6 expression may serve as a potential prognostic biomarker in BRCA.

### 3.2 PRDX6 Promoted Proliferation, Migration, and Invasion In Vitro

**FIGURE 2:**
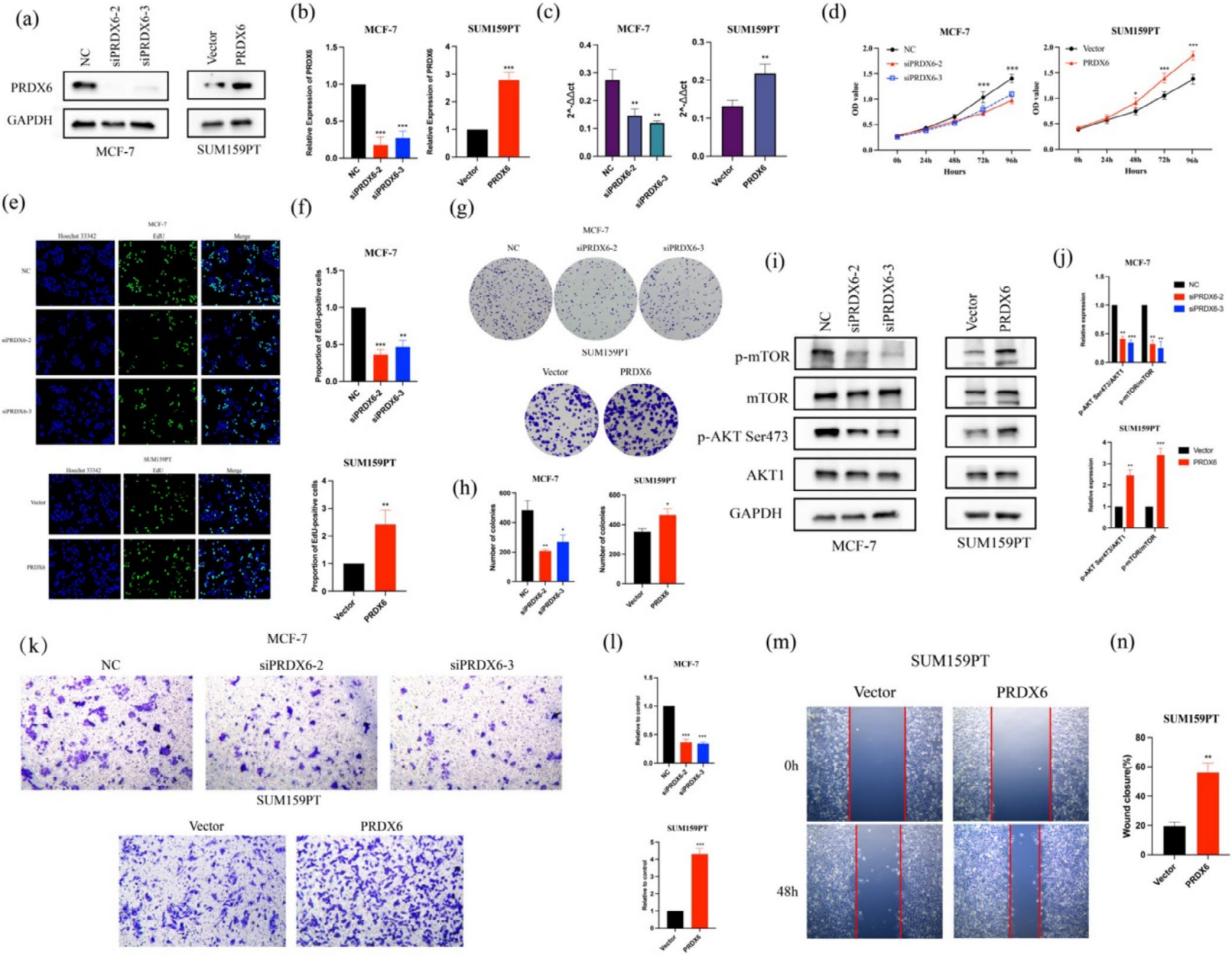
PRDX6 promoted cell proliferation, migration, and invasion in vitro. (a–c) Validation of knockdown and overexpression effects of PRDX6 protein (a, b) and mRNA (c). (d–h) CCK8 assay (d), EdU staining (e, f), and colony formation assay (g, h) were used to evaluate the effect of PRDX6 on cell proliferation. The results showed that knockdown of RDDX6 inhibited the proliferative capacity of MCF‐7, and PRDX6 overexpression promoted the proliferative capacity of SUM159PT cells in vitro. (i, j) Overexpression of PRDX6 led to increased phosphorylation levels of mTOR and AKT at Ser473, and the opposite phenomenon was observed when PRDX6 was knocked down. (k, l) Knockdown of PRDX6 inhibited the invasive ability of MCF‐7 cells, while overexpression of PRDX6 enhanced the invasiveness of SUM159PT cells. (m, n) Overexpression of PRDX6 promotes migration of SUM159PT cells. **p < 0.01; ***p < 0.001.

We investigated the expression levels of PRDX6 in six BRCA cell lines (MDA‐MB‐231, BT549, SUM159PT, MCF‐7, T47D, and SKBR3). Our findings indicated that PRDX6 was expressed at the highest level in MCF‐7 and T47D cells and at the lowest level in BT‐549 and SUM159PT cells (Figure S2). Therefore, we choose to knock down PRDX6 in MCF‐7 cells and overexpress PRDX6 in SUM159PT cells to explore its biological effects in BRCA cells. Lentiviral particles containing PRDX6 oligonucleotides were utilized to overexpress PRDX6 in SUM159PT cells (PRDX6), while siRNA targeting PRDX6 was employed to silence PRDX6 expression in MCF‐7 cells (si‐PRDX6). SiPRDX6‐2 and siPRDX6‐3, which had the best knockdown effect, were selected for subsequent experiments (Figure S3). The overexpression and silencing of PRDX6 were verified by western blotting and RT‐qPCR (Figure 2a–c).

To evaluate the effect of PRDX6 on cell proliferation, we conducted CCK‐8 assay, EdU staining, and colony formation assays. Overexpression of PRDX6 significantly enhanced the proliferative capacity and colony‐forming ability of SUM159PT cells compared to the control group (Figure 2d–h). Conversely, silencing PRDX6 in MCF‐7 cells yielded opposite results, demonstrating reduced proliferation and colony formation (Figure 2d–h). Moreover, PRDX6 overexpression increased the phosphorylation levels of AKT at Ser473 and mTOR without altering the expression levels of AKT1 and mTOR (Figure 2i,j).

We then assessed the influence of PRDX6 on cell migration and invasion by wound‐healing and Matrigel‐coated transwell assays. PRDX6 overexpression in SUM159PT cells led to increased migration and invasion capabilities (Figure 2k–n). In contrast, PRDX6 silencing resulted in diminished invasion in MCF‐7 cells (Figure 2k,l). These findings highlight the role of PRDX6 in promoting malignancy in BRCA cells in vitro.

**FIGURE 3:**
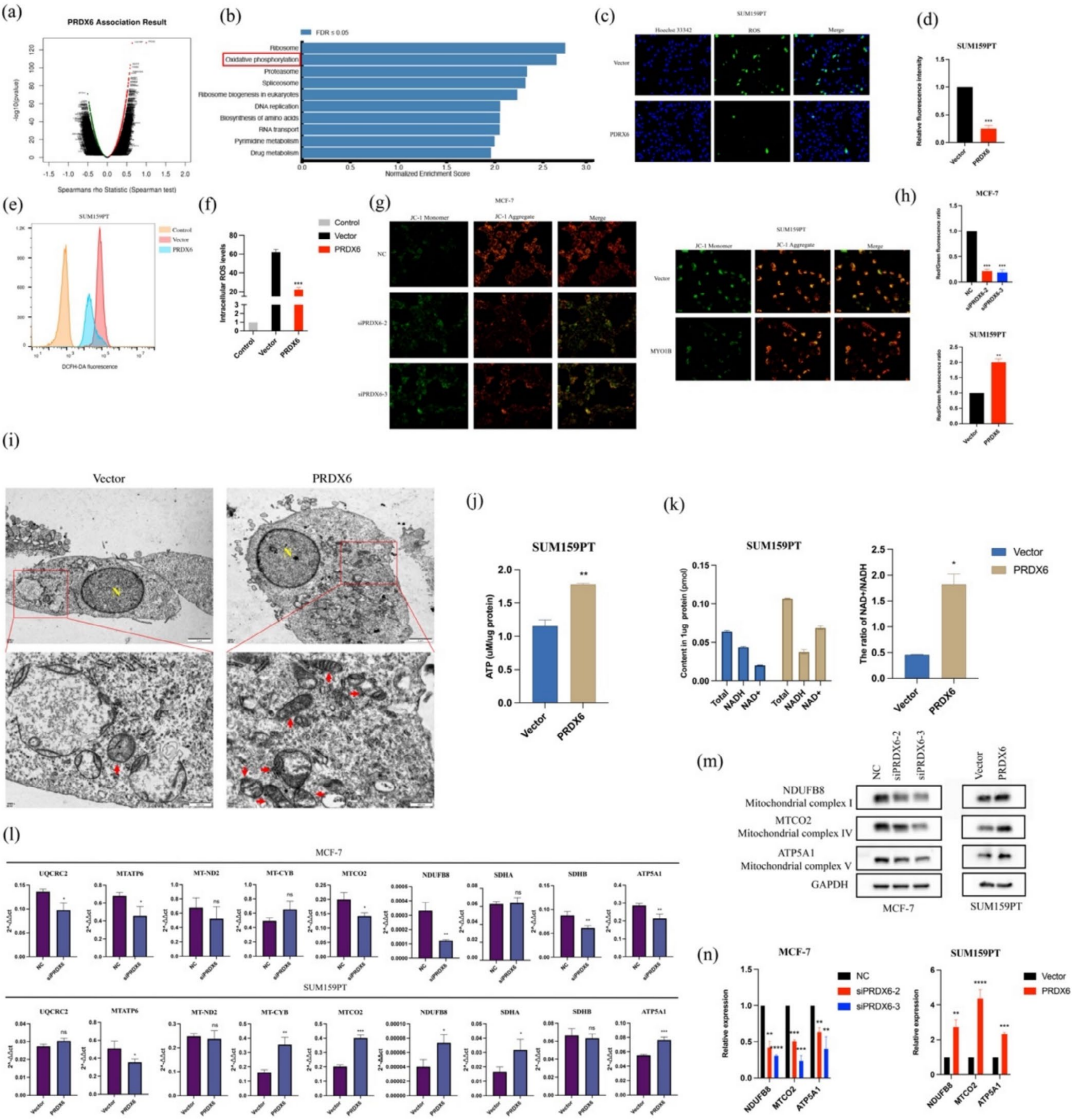
PRDX6 promoted mitochondrial biosynthesis and OXPHOS. (a) Heatmap of PRDX6‐associated genes generated using the LinkedOmics database. (b) Genes associated with PRDX6 were enriched in the OXPHOS pathway (FDR < 0.05). (c–f) Fluorescence microscopy (c, d) and flow cytometry (e, f) were used to assess intracellular ROS levels. (g, h) The mitochondrial membrane potential levels in cells were evaluated using fluorescence microscopy. (i) In the vector group, mitochondria were fewer, swollen, with sparse matrix, dissolved contents, and reduced cristae. In the PRDX6 overexpression group, mitochondria increased, with clear double membranes, lamellar cristae, and parallel arrangement. Red arrows: Mitochondria; N: Nucleus. Top: 2500×; bottom: 10,000×. (j) PRDX6 promoted intracellular ATP production. (k) PRDX6 overexpression led to an elevated intracellular NAD+/NADH ratio. (l) Regulation of mitochondrial complex subunit mRNA expression levels by PRDX6 expression. (m, n) PRDX6 regulated protein expression of mitochondrial complex subunits NDUFB8, MTCO2 and ATP5A1. **p < 0.01; ***p < 0.001.

### 3.3 PRDX6 Enhanced Mitochondrial Function and OXPHOS in BRCA Cells

To investigate the mechanism of PRDX6‐mediated progression in BRCA, we identified genes associated with PRDX6 expression using RNA‐seq data of TCGA_BRCA from the LinkedOmics platform [12]. A total of 13,705 genes were identified, of which 5648 were positively and 8057 negatively associated with PRDX6 (*p* < 0.05, Figure 3a; Table S2 for a complete list of genes). KEGG enrichment analysis of these genes showed that the top three enriched pathways were ribosomes, OXPHOS, and proteasome (FDR < 0.05, Figure 3b; Table S3 for a complete list of pathways). We analyzed the expression of PRDX family members and key subunits of the mitochondrial complex in the OXPHOS system, and found that the majority of these genes exhibited a strong positive correlation with PRDX6 expression in BRCA (*r* > 0.3, *p* < 0.05, Table 1).

**TABLE 1:** Expression correlation of PRDX family and OXPHOS complex subunits with PRDX6 in BRCA.

Genes	*r*	*p*
PRDX family members		
PRDX1	0.35	4.18E‐32
PRDX2	0.20	7.47E‐11
PRDX4	0.43	6.61E‐51
PRDX5	0.23	1.24E‐14
Complex I		
NDUFB3	0.32	1.68E‐27
NDUFB9	0.39	3.05E‐40
NDUFA1	0.37	9.15E‐38
NDUFB5	0.32	2.48E‐27
NDUFV3	0.40	7.65E‐43
NDUFAB1	0.35	1.33E‐33
Complex II		
SDHC	0.42	1.82E‐47
Complex III		
UQCRB	0.41	1.49E‐44
UQCRH	0.37	3.08E‐37
Complex IV		
COX5A	0.37	7.09E‐36
COX5B	0.33	1.34E‐28
COX6A1	0.32	6.68E‐27
COX6B1	0.42	5.00E‐49
COX7A2	0.30	1.16E‐24
Complex V		
ATP5C1	0.44	3.91E‐54
ATP5E	0.37	1.73E‐37
ATP5J	0.39	2.46E‐40
ATP5O	0.38	1.16E‐38
ATP5G1	0.36	5.96E‐35
ATP5G3	0.40	8.60E‐43

As previously reported, we found that overexpression of PRDX6 scavenged ROS in BRCA cells (Figure 3c–f). We also found that mitochondrial membrane potential was elevated after overexpression of PRDX6 and decreased after silencing of PRDX6 (Figure 3g,h). What is more, transmission electron microscopy (TEM) showed that cells overexpressing PRDX6 exhibited an increased number of mitochondria, featuring clearly defined double membranes, visible lamellar cristae, and roughly parallel‐aligned tubules, suggestive of active mitochondrial synthesis and function, as compared with controls (Figure 3i). Overexpression of PRDX6 also resulted in increased intracellular ATP production (Figure 3j) and higher NAD^+^/NADH ratios (Figure 3k), suggesting an active OXPHOS process.

### 3.4 PRDX6 Promoted Mitochondrial Biosynthesis

While it is well‐established that PRDX6 can mitigate oxidative stress‐induced mitochondrial damage through its unique iPLA2 and GPx activities, our results indicated that PRDX6 may also induce mitochondria into an active state of biogenesis and OXPHOS to meet the high energy demands of cancer cells. Therefore, we sought to explore whether PRDX6 promotes the synthesis of mitochondrial complexes to maintain mitochondrial function.

As expected, knockdown of PRDX6 led to a reduction in the mRNA levels of most mitochondrial complex subunits, including NDUFB8 (Complex I), SDHB (Complex II), UQCRC2 (Complex III), MTCO2 (Complex IV), and MTATP6/ATP5A1 (Complex V). Conversely, overexpression of PRDX6 resulted in the upregulation of SDHA, NDUFB8, MT‐CYB (Complex III), MTCO2, and ATP5A1 expression (Figure 3l). We selected NDUFB8, MTCO2, and ATP5A1, which showed consistent trends in mRNA expression changes across two cell lines, for western blotting validation. The results demonstrated that the protein levels and their changes were consistent with the corresponding mRNA levels (Figure 3m,n). These findings suggested that PRDX6 can influence the expression of at least a subset of mitochondrial matrix complex subunits.

### 3.5 PRDX6 Regulated the Mitochondrial Biosynthesis Through TFAM‐Dependent and ‐Independent Pathways

**FIGURE 4:**
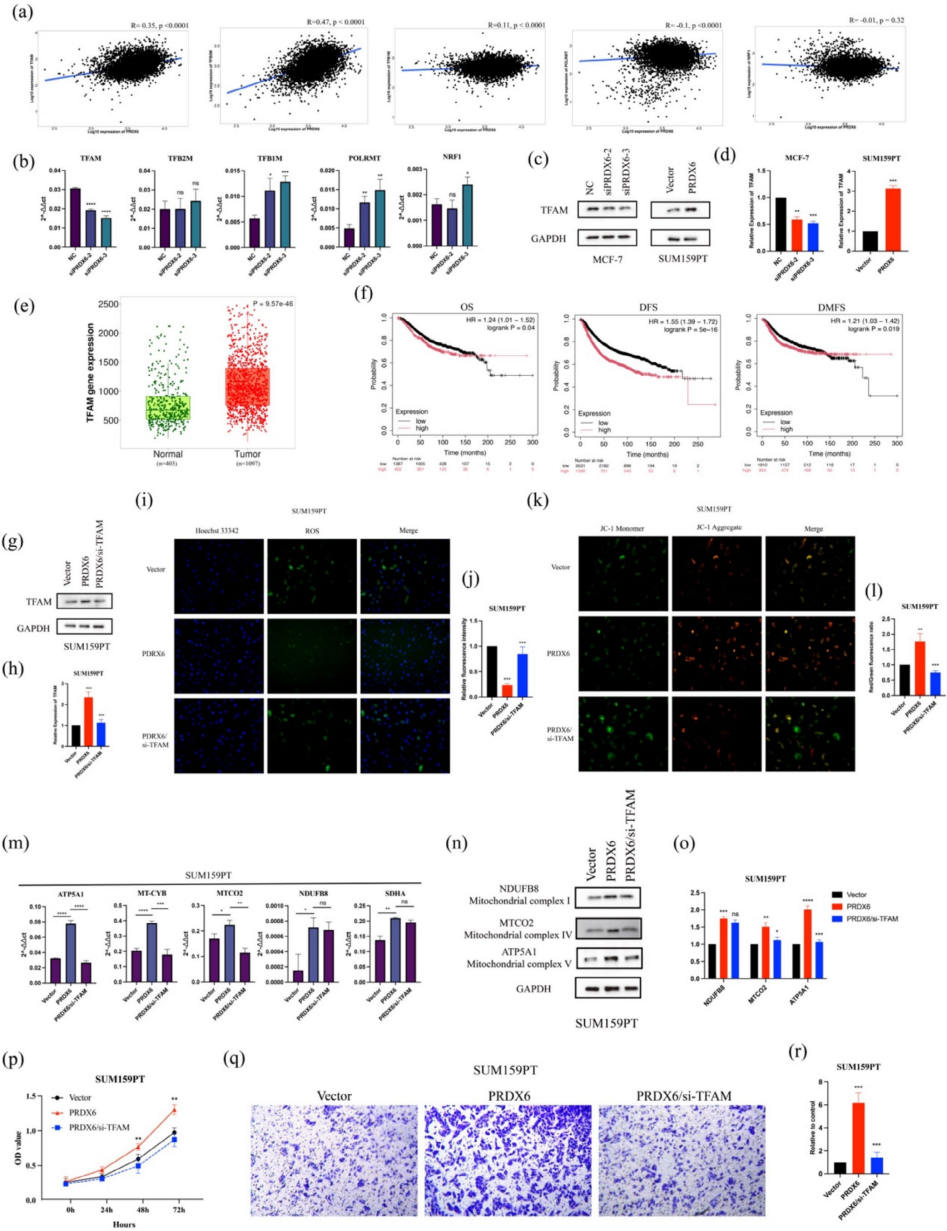
TFAM was a target of PRDX6. (a) PRDX6 expression was positively correlated with TFAM (*r* = 0.35, *p* < 0.0001) and TFB2M (*r* = 0.47, *p* < 0.0001) expression. (b) PRDX6 affected the mRNA expression level of TFAM. (c, d) PRDX6 affected the protein expression level of TFAM. (e) TFAM was highly expressed in BRCA. (f) TFAM was associated with worse OS, DFS, and DMFS in BRCA patients. (g, h) siRNA against TFAM knocked down the expression level of TFAM. (i–l) Knockdown of TFAM increased ROS production (i, j) and reduced the mitochondrial membrane potential (k, l). (m) Knockdown of TFAM reduced the expression of ATP5A1, MT‐CYB, and MTCO2 caused by PRDX6 overexpression. (n, o) Knockdown of TFAM reduced the synthesis of MTCO2 and ATP5A1 caused by PRDX6 overexpression. (p, r) Enhanced proliferation (p) and invasive capacity (q,r) Induced by PRDX6 was suppressed after knockdown of TFAM. *p < 0.05; **p < 0.01; ***p < 0.001.

To further elucidate the potential mechanism by which PRDX6 promoted mitochondrial biogenesis, we analyzed the correlation between PRDX6 and several key transcription factors and coactivators involved in mitochondrial biogenesis using the TNMplot database. These factors included POLRMT (Polymerase (RNA) Mitochondrial, DNA directed), NRF1 (Nuclear Respiratory Factor 1), TFB1M (Transcription Factor B1, Mitochondrial), TFB2M (Transcription Factor B2, Mitochondrial), and TFAM (Mitochondrial transcription factor A). The results revealed a significant positive correlation between PRDX6 expression and TFAM (*r* = 0.35, *p* < 0.0001) as well as TFB2M (*r* = 0.47, *p* < 0.0001) in BRCA tissues (Figure 4a). RT‐qPCR showed that only TFAM expression decreased after the knockdown of PRDX6 (Figure 4b). Western blotting further confirmed that the expression of TFAM proteins changed synchronously with changes in PRDX6 expression (Figure 4c,d).

TFAM, also known as mtTFA, is an important mitochondrial transcription factor responsible for regulating the transcription and replication of the 13 genes encoded by mitochondrial DNA (mtDNA) and promoting the synthesis of mitochondrial matrix proteins. We found that TFAM expression was upregulated in BRCA tissues (Figure 4e) and was associated with worse OS, DFS, and DMFS in patients (Figure 4f). We then transfected siRNA targeting TFAM into SUM159PT cells stably overexpressing PRDX6 (Figure 4g,h) and found that silencing of TFAM led to an increase in intracellular ROS generation and a decrease in mitochondrial membrane potential (Figure 4i–l), suggesting that mitochondrial function was inhibited. However, knockdown of TFAM only reduced the mRNA levels of ATP5A1, MT‐CYB, and MTCO2 without altering the elevated expression of NDUFB8 and SDHA caused by PRDX6 overexpression (Figure 4m). Western blotting also demonstrated that knockdown of TFAM resulted in no significant changes in NDUFB8 expression and reduced synthesis of MTCO2 and ATP5A1 (Figure 4n,o). Finally, we found that the enhanced proliferation (Figure 4p) and invasive capacity (Figure 4o–r) of cells induced by PRDX6 was suppressed after knockdown of TFAM.

### 3.6 PRDX6 Promoted Tumor Growth In Vivo

**FIGURE 5:**
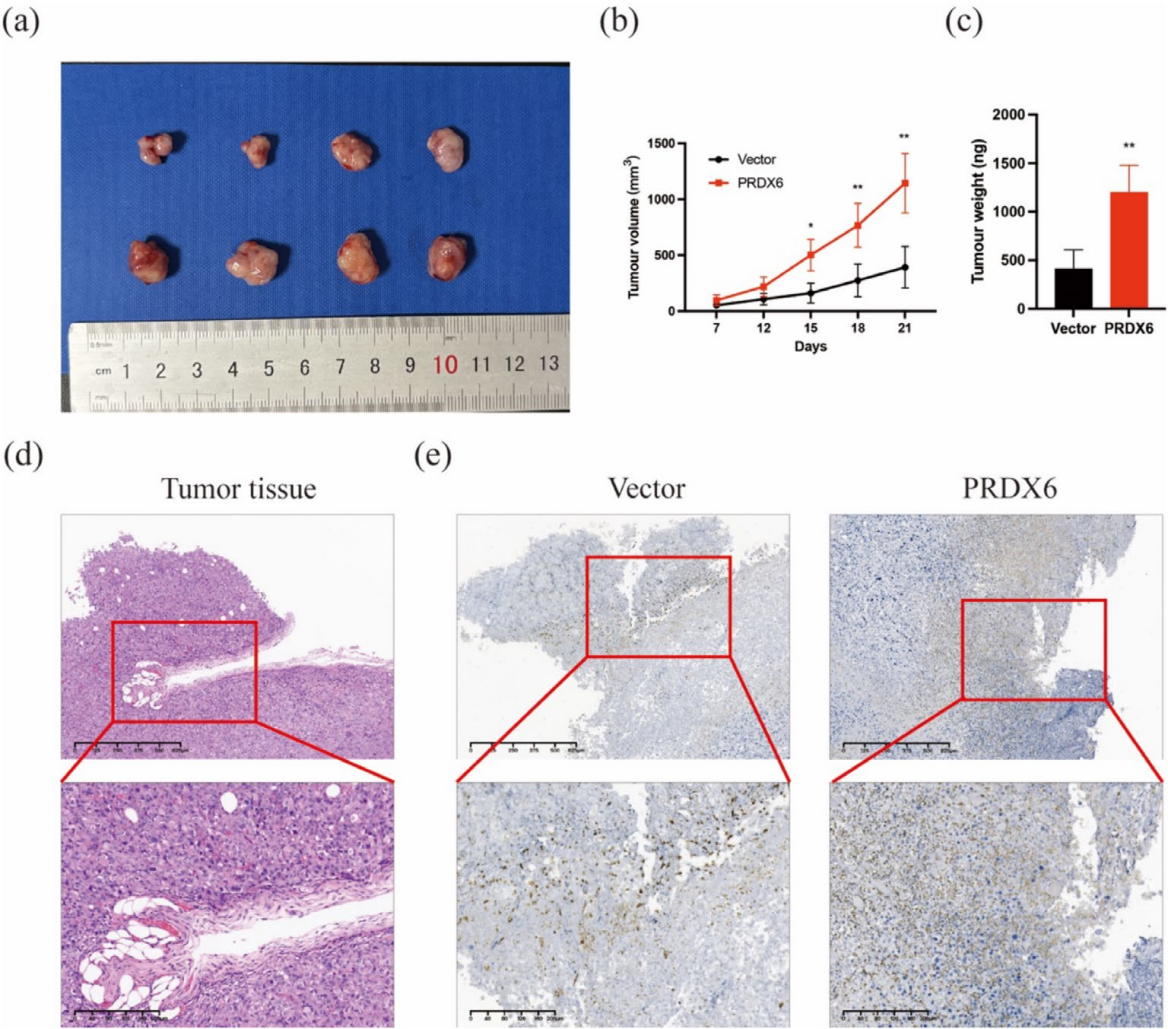
PRDX6 promoted tumor growth in vivo. (a–c) Compared with the control group, the tumors in the PRDX6 overexpression group of xenograft model mice were larger in size (a, b) and heavier in mass (c); Top: 4×; bottom: 10×. (d) HE staining sections of tumor tissue. (e) Representative IHC staining of Ki67 in tumor tissues from the control and PRDX6 overexpression groups; Top: 4×; bottom: 10×. *p < 0.05; **p < 0.01. HE, Hematoxylin–eosin; IHC, immunohistochemical staining.

Finally, we established an SUM159PT cell xenograft model with stable overexpression of PRDX6 in immunodeficient mice. Tumor growth was monitored every three to four days over a three‐week period. After three weeks, the mice were euthanized, and the tumors were weighed. Compared to the control group, overexpression of PRDX6 significantly increased both tumor volume and weight (Figure 5a–c). Figure 5d shows Hematoxylin–eosin (HE) stained sections of tumor tissue. Additionally, immunohistochemical staining (IHC) revealed a marked increase in Ki67 expression in PRDX6‐overexpressing tumors (Figure 5e). These results were consistent with the in vitro findings, indicating that PRDX6 significantly promotes tumor growth in vivo.

## 4 Discussion

BRCA, as a systemic disease, is treated with endocrine therapy, chemotherapy, targeted therapy, immunotherapy, and radiotherapy, in addition to surgery [2, 13, 14]. This means that tumor cells are inevitably exposed to oxidative stress for a long period of time. Although the role of PRDX6 in protecting cells from oxidative stress has been elucidated in a variety of tumors [7, 15, 16, 17], its relationship with BRCA has not been fully investigated.

PRDX6 expression in BRCA has been examined in previous studies, with Karihtala et al. highlighting its overexpression in ER+ BRCA cases [10]. Additionally, PRDX6 is highly expressed in drug‐resistant BRCA cell lines, potentially linking it to chemotherapy resistance [9, 11]. Conversely, some studies report lower PRDX6 levels in BRCA tissues than in normal tissues, yet higher expression correlates with worse prognosis in BRCA patients [18]. These inconsistent findings suggest that the role PRDX6 serves in BRCA still needs to be explored in depth.

We demonstrated that PRDX6 expression is lower in BRCA tissues compared to normal breast tissues. However, PRDX6 exhibited subtype‐specific overexpression in more aggressive BRCA subtypes and advanced‐stage tumors, and its high expression was significantly associated with poor prognosis. We hypothesize that this “subtype‐specific overexpression” may have been masked in analyses of the entire cohort due to averaging effects, yet the oncogenic role of PRDX6 still adversely impacts prognosis. Further functional assays confirmed that PRDX6 significantly promoted proliferation, migration, and invasion in both ER‐positive BRCA cells (MCF‐7) and TNBC cells (SUM159PT), and significantly accelerated tumor growth in vivo, supporting its oncogenic role irrespective of BRCA subtype.

Most studies have attributed the oncogenic role of PRDX6 to its dual enzymatic activities as GPx and iPLA2 [7, 16, 19, 20]. ROS exert cytotoxic effects on cancer cells, while PRDX6 detoxifies ROS and maintains cellular homeostasis [21, 22]. In contrast, PRDX6 deficiency leads to lipid peroxidation and ROS accumulation [23], which can activate various signaling pathways, including ferroptosis, ultimately resulting in cancer cell death [24]. Notably, a recent study by Wu et al. revealed a nonenzymatic mechanism of PRDX6‐mediated tumor promotion, wherein PRDX6 prevents the ubiquitination and degradation of nicotinamide N‐methyltransferase (NNMT), thereby activating the MAPK signaling pathway and facilitating ovarian cancer progression [25]. In this study, we found that PRDX6 robustly promotes mitochondrial biogenesis and OXPHOS, as evidenced by increased mitochondrial membrane potential, elevated ATP production and NAD^+^/NADH ratio, an increased number and cristae density of mitochondria, and upregulation of most mitochondrial complex subunits.

The role of OXPHOS in the progression of malignancy has garnered escalating scrutiny in recent years. Although the majority of tumors manifest the ‘Warburg effect’, a phenomenon wherein neoplastic cells preferentially engage in aerobic glycolysis for energy generation even in oxygen‐rich environments [26], emerging data indicate that OXPHOS persists in its activity within cancer cells, especially during the stages of tumorigenesis, chemoresistance, and metastasis [27, 28, 29]. Integrative multi‐omics analyses have revealed that certain metastatic ER+ BRCA exhibit a pronounced reliance on OXPHOS [30]. A separate study has also indicated that TNBC patients who exhibit diminished responsiveness following neoadjuvant chemotherapy display an enhanced OXPHOS signature [31]. Consequently, tumor cells may reactivate mitochondrial function to accommodate their escalating bioenergetic demands, thereby underscoring the metabolic plasticity of cancer cells and their capacity to adapt to diverse microenvironmental stresses.

Through integrated analysis of public databases and experimental validation, we identified TFAM as a potential mediator of PRDX6‐regulated mitochondrial function. TFAM, the primary mitochondrial transcription factor, is transcribed in the nucleus and translated into a precursor protein in the cytoplasm [32, 33]. It is then imported into mitochondria via its N‐terminal mitochondrial targeting sequence, where it matures and regulates the replication and transcription of the 13 genes encoded by mtDNA [34]. Previous studies have shown that TFAM knockdown in BRCA cells enhances sensitivity to cisplatin [35, 36], suggesting that TFAM may play a pro‐cancer role in BRCA. In our study, we found that TFAM is highly expressed in BRCA and is significantly associated with poor prognosis. PRDX6 upregulates both mRNA and protein levels of TFAM, and TFAM knockdown in PRDX6‐overexpressing cells reversed the upregulation of mitochondrial complex subunits (including ATP5A1, MT‐CYB, and MTCO2) and the enhanced proliferative and invasive phenotypes induced by PRDX6. This phenomenon may be related to the inhibitory effects of ROS on cellular transcription and translation. ROS accumulation can cause genomic DNA damage [37, 38], activate DNA damage repair pathways, and lead to cell cycle arrest and transcriptional suppression [39, 40, 41]. Additionally, ROS can induce endoplasmic reticulum (ER) stress, triggering the unfolded protein response (UPR) and impairing protein synthesis and folding [42, 43, 44]. We hypothesize that PRDX6 maintained intracellular homeostasis by scavenging ROS in both the cytoplasm and mitochondria, thereby promoting the transcription and translation of TFAM and nuclear‐encoded mitochondrial subunits. This, in turn, enhanced mitochondrial biogenesis and OXPHOS to meet the high energy demands of cancer cells.

## 5 Conclusion

This study highlighted the critical role of PRDX6 in BRCA progression, particularly its ability to promote mitochondrial biogenesis and OXPHOS through both TFAM‐dependent and ‐independent pathways, thereby enhancing cancer cell proliferation, migration, and invasion. Our findings suggest that PRDX6 may serve as a potential target in BRCA, offering new insights for future diagnostic and therapeutic strategies.

## Author Contributions

**Mei Dai:** writing – original draft, investigation, methodology, validation, software, formal analysis. **Danhua Zhang:** conceptualization, writing – review and editing, validation, project administration, supervision.

## Ethics Statement

The study is reported in accordance with ARRIVE guidelines **(** https://arriveguidelines.org). All animal experiments followed the regulations of the Animal Ethical and Welfare Committee of the Second Xiangya Hospital (Approval No. 20241090).

## Conflicts of Interest

The authors declare no conflicts of interest.

## Supporting information


**Figures S1–S3**.

**Table S1**.

**Table S2**.

**Table S3**.

## Data Availability

The data that support the findings of this study are available on request from the corresponding author. The data are not publicly available due to privacy or ethical restrictions.

## References

[1] R. L. Siegel , K. D. Miller , H. E. Fuchs , and A. Jemal , “Cancer Statistics, 2022,” CA: A Cancer Journal for Clinicians 72 (2022): 7–33. 35020204 10.3322/caac.21708

[2] N. Harbeck and M. Gnant , “Breast Cancer,” Lancet 389 (2017): 1134–1150. 27865536 10.1016/S0140-6736(16)31891-8

[3] A MB, R VS, J ME, A AO , “Breast Cancer Biomarkers: Risk Assessment, Diagnosis, Prognosis, Prediction of Treatment Efficacy and Toxicity, and Recurrence,” Current Pharmaceutical Design 20 (2014): 4879–4898. 24283956 10.2174/1381612819666131125145517

[4] S. Rajput , P. K. Sharma , and R. Malviya , “Biomarkers and Treatment Strategies for Breast Cancer Recurrence,” Current Drug Targets 24 (2023): 1209–1220. 38164731 10.2174/0113894501258059231103072025

[5] H. Rahaman , K. Herojit , L. R. Singh , R. Haobam , and A. B. Fisher , “Structural and Functional Diversity of the Peroxiredoxin 6 Enzyme Family,” Antioxidants & Redox Signaling 40 (2024): 759–775. 37463006 10.1089/ars.2023.0287

[6] X. Hu , E. Lu , C. Pan , Y. Xu , and X. Zhu , “Overexpression and Biological Function of PRDX6 in Human Cervical Cancer,” Journal of Cancer 11 (2020): 2390–2400. 32201510 10.7150/jca.39892 PMC7066013

[7] H. M. Yun , K. R. Park , H. P. Lee , et al., “PRDX6 Promotes Lung Tumor Progression via Its GPx and iPLA2 Activities,” Free Radical Biology & Medicine 69 (2014): 367–376. 24512906 10.1016/j.freeradbiomed.2014.02.001

[8] W. S. Huang , C. Y. Huang , M. C. Hsieh , et al., “Expression of PRDX6 Correlates With Migration and Invasiveness of Colorectal Cancer Cells,” Cellular Physiology and Biochemistry 51 (2018): 2616–2630. 30562740 10.1159/000495934

[9] X. Z. Chang , D. Q. Li , Y. F. Hou , et al., “Identification of the Functional Role of Peroxiredoxin 6 in the Progression of Breast Cancer,” Breast Cancer Research 9 (2007): R76. 17980029 10.1186/bcr1789 PMC2246172

[10] P. Karihtala , A. Mäntyniemi , S. W. Kang , V. L. Kinnula , and Y. Soini , “Peroxiredoxins in Breast Carcinoma,” Clinical Cancer Research 9 (2003): 3418–3424. 12960131

[11] Y. Liu , H. Liu , B. Han , and J. T. Zhang , “Identification of 14‐3‐3sigma as a Contributor to Drug Resistance in Human Breast Cancer Cells Using Functional Proteomic Analysis,” Cancer Research 66 (2006): 3248–3255. 16540677 10.1158/0008-5472.CAN-05-3801

[12] S. V. Vasaikar , P. Straub , J. Wang , and B. Zhang , “LinkedOmics: Analyzing Multi‐Omics Data Within and Across 32 Cancer Types,” Nucleic Acids Research 46 (2018): D956–D963. 29136207 10.1093/nar/gkx1090 PMC5753188

[13] R. A. Leon‐Ferre and M. P. Goetz , “Advances in Systemic Therapies for Triple Negative Breast Cancer,” BMJ (Clinical Research Ed.) 381 (2023): e071674. 10.1136/bmj-2022-071674 37253507

[14] E. S. McDonald , A. S. Clark , J. Tchou , P. Zhang , and G. M. Freedman , “Clinical Diagnosis and Management of Breast Cancer,” Journal of Nuclear Medicine 57, no. Suppl 1 (2016): 9s–16s. 26834110 10.2967/jnumed.115.157834

[15] Y. He , W. Xu , Y. Xiao , et al., “Overexpression of Peroxiredoxin 6 (PRDX6) Promotes the Aggressive Phenotypes of Esophageal Squamous Cell Carcinoma,” Journal of Cancer 9 (2018): 3939–3949. 30410598 10.7150/jca.26041 PMC6218759

[16] M. Jo , H. M. Yun , K. R. Park , et al., “Lung Tumor Growth‐Promoting Function of Peroxiredoxin 6,” Free Radical Biology & Medicine 61 (2013): 453–463. 23643677 10.1016/j.freeradbiomed.2013.04.032

[17] H. M. Yun , K. R. Park , M. H. Park , et al., “PRDX6 Promotes Tumor Development via the JAK2/STAT3 Pathway in a Urethane‐Induced Lung Tumor Model,” Free Radical Biology & Medicine 80 (2015): 136–144. 25582888 10.1016/j.freeradbiomed.2014.12.022

[18] G. Wang , W. C. Zhong , Y. H. Bi , et al., “The Prognosis of Peroxiredoxin Family in Breast Cancer,” Cancer Management and Research 11 (2019): 9685–9699. 31814764 10.2147/CMAR.S229389 PMC6861534

[19] D. J. Lagal , M. J. López‐Grueso , J. R. Pedrajas , et al., “Loss of PRDX6 Aborts Proliferative and Migratory Signaling in Hepatocarcinoma Cell Lines,” Antioxidants 12, no. 6 (2023): 1153. 37371884 10.3390/antiox12061153 PMC10294896

[20] M. J. López‐Grueso , D. J. Lagal , Á. F. García‐Jiménez , et al., “Knockout of PRDX6 Induces Mitochondrial Dysfunction and Cell Cycle Arrest at G2/M in HepG2 Hepatocarcinoma Cells,” Redox Biology 37 (2020): 101737. 33035814 10.1016/j.redox.2020.101737 PMC7554216

[21] H. Melhem , M. R. Spalinger , J. Cosin‐Roger , et al., “Prdx6 Deficiency Ameliorates DSS Colitis: Relevance of Compensatory Antioxidant Mechanisms,” Journal of Crohn's & Colitis 11 (2017): 871–884. 10.1093/ecco-jcc/jjx016 28199527

[22] G. Y. Liu , J. X. Shi , S. L. Shi , et al., “Nucleophosmin Regulates Intracellular Oxidative Stress Homeostasis via Antioxidant PRDX6,” Journal of Cellular Biochemistry 118 (2017): 4697–4707. 28513872 10.1002/jcb.26135

[23] D. J. Lagal , A. M. Montes‐Osuna , A. Ortiz‐Olivencia , et al., “Tumoral Malignancy Decreases Coupled With Higher ROS and Lipid Peroxidation in HCT116 Colon Cancer Cells Upon Loss of PRDX6,” Antioxidants (Basel) 13, no. 7 (2024): 881. 39061949 10.3390/antiox13070881 PMC11274330

[24] J. Ito , T. Nakamura , T. Toyama , et al., “PRDX6 Dictates Ferroptosis Sensitivity by Directing Cellular Selenium Utilization,” Molecular Cell 84 (2024): 4629–44.e9. 39547222 10.1016/j.molcel.2024.10.028

[25] X. Wu , L. Luo , M. Wang , et al., “PRDX6 Prevents NNMT Ubiquitination and Degradation as a Nonenzymatic Mechanism to Promote Ovarian Cancer Progression,” Advanced Science 12 (2025): e2416484. 39887931 10.1002/advs.202416484 PMC11948025

[26] A. Fukushi , H. D. Kim , Y. C. Chang , and C. H. Kim , “Revisited Metabolic Control and Reprogramming Cancers by Means of the Warburg Effect in Tumor Cells,” International Journal of Molecular Sciences 23 (2022): 10037. 36077431 10.3390/ijms231710037 PMC9456516

[27] T. M. Ashton , W. G. McKenna , L. A. Kunz‐Schughart , and G. S. Higgins , “Oxidative Phosphorylation as an Emerging Target in Cancer Therapy,” Clinical Cancer Research 24 (2018): 2482–2490. 29420223 10.1158/1078-0432.CCR-17-3070

[28] J. Greene , A. Segaran , and S. Lord , “Targeting OXPHOS and the Electron Transport Chain in Cancer; Molecular and Therapeutic Implications,” Seminars in Cancer Biology 86 (2022): 851–859. 35122973 10.1016/j.semcancer.2022.02.002

[29] B. Kalyanaraman , G. Cheng , M. Hardy , and M. You , “OXPHOS‐Targeting Drugs in Oncology: New Perspectives,” Expert Opinion on Therapeutic Targets 27 (2023): 939–952. 37736880 10.1080/14728222.2023.2261631 PMC11034819

[30] R. El‐Botty , L. Morriset , E. Montaudon , et al., “Oxidative Phosphorylation Is a Metabolic Vulnerability of Endocrine Therapy and Palbociclib Resistant Metastatic Breast Cancers,” Nature Communications 14 (2023): 4221. 10.1038/s41467-023-40022-5 PMC10349040 37452026

[31] K. W. Evans , E. Yuca , S. S. Scott , et al., “Oxidative Phosphorylation Is a Metabolic Vulnerability in Chemotherapy‐Resistant Triple‐Negative Breast Cancer,” Cancer Research 81 (2021): 5572–5581. 34518211 10.1158/0008-5472.CAN-20-3242 PMC8563442

[32] R. C. Scarpulla , “Transcriptional Paradigms in Mammalian Mitochondrial Biogenesis and Function,” Physiological Reviews 88 (2008): 611–638. 18391175 10.1152/physrev.00025.2007

[33] N. Kozhukhar and M. F. Alexeyev , “35 Years of TFAM Research: Old Protein, New Puzzles,” Biology 12, no. 6 (2023): 823. 37372108 10.3390/biology12060823 PMC10295803

[34] Y. I. Morozov , K. Agaronyan , A. C. Cheung , M. Anikin , P. Cramer , and D. Temiakov , “A Novel Intermediate in Transcription Initiation by Human Mitochondrial RNA Polymerase,” Nucleic Acids Research 42 (2014): 3884–3893. 24393772 10.1093/nar/gkt1356 PMC3973326

[35] X. Fan , S. Zhou , M. Zheng , X. Deng , Y. Yi , and T. Huang , “MiR‐199a‐3p Enhances Breast Cancer Cell Sensitivity to Cisplatin by Downregulating TFAM (TFAM),” Biomedicine & Pharmacotherapy 88 (2017): 507–514. 28126676 10.1016/j.biopha.2017.01.058

[36] W. Gao , M. H. Wu , N. Wang , et al., “Mitochondrial Transcription Factor A Contributes to Cisplatin Resistance in Patients With Estrogen Receptor‐Positive Breast Cancer,” Molecular Medicine Reports 14 (2016): 5304–5310. 27779689 10.3892/mmr.2016.5881

[37] S. Kawanishi , S. Ohnishi , N. Ma , Y. Hiraku , and M. Murata , “Crosstalk Between DNA Damage and Inflammation in the Multiple Steps of Carcinogenesis,” International Journal of Molecular Sciences 18 (2017): 1808. 28825631 10.3390/ijms18081808 PMC5578195

[38] A. S. Levine , L. Sun , R. Tan , et al., “The Oxidative DNA Damage Response: A Review of Research Undertaken With Tsinghua and Xiangya Students at the University of Pittsburgh,” Science China. Life Sciences 60 (2017): 1077–1080. 29067646 10.1007/s11427-017-9184-6

[39] A. Dutta , C. Yang , S. Sengupta , S. Mitra , and M. L. Hegde , “New Paradigms in the Repair of Oxidative Damage in Human Genome: Mechanisms Ensuring Repair of Mutagenic Base Lesions During Replication and Involvement of Accessory Proteins,” Cellular and Molecular Life Sciences 72 (2015): 1679–1698. 25575562 10.1007/s00018-014-1820-z PMC4395533

[40] B. Li , P. Zhou , K. Xu , et al., “Metformin Induces Cell Cycle Arrest, Apoptosis and Autophagy Through ROS/JNK Signaling Pathway in Human Osteosarcoma,” International Journal of Biological Sciences 16 (2020): 74–84. 31892847 10.7150/ijbs.33787 PMC6930379

[41] M. D. Kuczler , A. M. Olseen , K. J. Pienta , and S. R. Amend , “ROS‐Induced Cell Cycle Arrest as a Mechanism of Resistance in Polyaneuploid Cancer Cells (PACCs),” Progress in Biophysics and Molecular Biology 165 (2021): 3–7. 33991583 10.1016/j.pbiomolbio.2021.05.002 PMC8511226

[42] Z. Zhang , L. Zhang , L. Zhou , Y. Lei , Y. Zhang , and C. Huang , “Redox Signaling and Unfolded Protein Response Coordinate Cell Fate Decisions Under ER Stress,” Redox Biology 25 (2019): 101047. 30470534 10.1016/j.redox.2018.11.005 PMC6859529

[43] Y. Lin , M. Jiang , W. Chen , T. Zhao , and Y. Wei , “Cancer and ER Stress: Mutual Crosstalk Between Autophagy, Oxidative Stress and Inflammatory Response,” Biomedicine & Pharmacotherapy 118 (2019): 109249. 31351428 10.1016/j.biopha.2019.109249

[44] R. Ozgur , B. Uzilday , Y. Iwata , N. Koizumi , and I. Turkan , “Interplay Between the Unfolded Protein Response and Reactive Oxygen Species: A Dynamic Duo,” Journal of Experimental Botany 69 (2018): 3333–3345. 29415271 10.1093/jxb/ery040

