# Supplementary figures and images for: PRDX6 Drives Breast Cancer Progression Through Mitochondrial Biosynthesis and Oxidative Phosphorylation
Source: Cancer Med. 2025 Jun 30;14(13):e71005. doi: 10.1002/cam4.71005 (PMC12207245; doi:10.1002/cam4.71005)

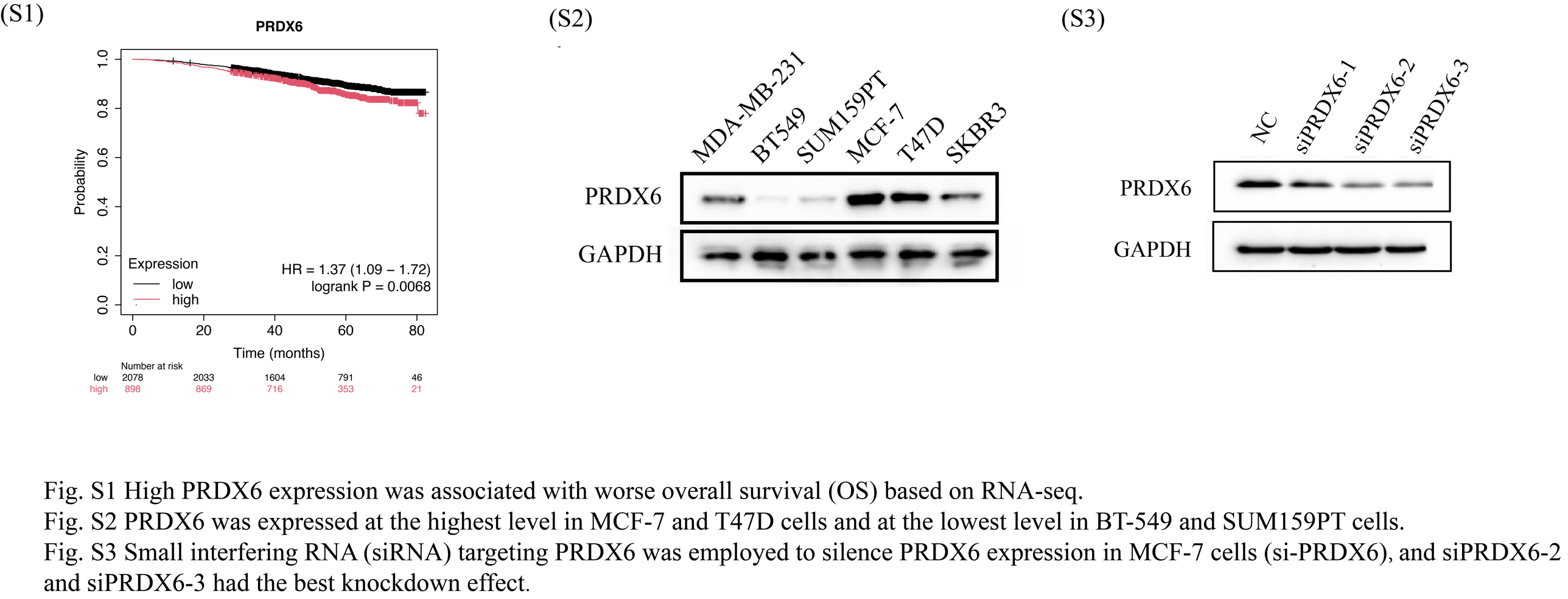

Supplement: Supplementary file 1 — Figures S1–S3. [file CAM4-14-e71005-s001.jpg]
