# Supplementary material for: PRDX6 Drives Breast Cancer Progression Through Mitochondrial Biosynthesis and Oxidative Phosphorylation
Source: Cancer Med. 2025 Jun 30;14(13):e71005. doi: 10.1002/cam4.71005 (PMC12207245; doi:10.1002/cam4.71005)
Supplement: Supplementary file 2 — Table S1. [file CAM4-14-e71005-s003.docx]

**Table S1. The qPCR primer sequences.**

| GAPDH | forward:5`-TGACCTGCCGTCTAGAAAAACCT-3` |
| --- | --- |
|  | reverse: 5`-GCTGTTGAAGTCAGAGGAGACCA-3` |
| PRDX6 | forward:5`- CTATCCTCTACCCAGCTACCAC -3` |
|  | reverse:5`- GGACCATCACACTATCCCCAT -3` |
| TFAM | forward:5`- ACTAATTAGAAGAATTGCCCAG -3` |
|  | reverse:5`- CTTCTTTATATACCTGCCACT -3` |
| NDUFB8 | forward:5`- TGGATACATCCCCCACACC-3` |
|  | reverse: 5`- AGCCTAAATTGTAAGAGAAGTGGT -3` |
| MT-ND2 | forward:5`- GCCCTAGAAATAAACATGCTA -3` |
|  | reverse: 5`- GGGCTATTCCTAGTTTTATT -3` |
| SDHA | forward:5`- TGGCCACTCGCTATTGCACAC -3` |
|  | reverse: 5`- GATGACACCACGGCACTCCC -3` |
| SDHB | forward:5`- CACTCTAGCTTGCACCCGAA -3` |
|  | reverse: 5`- CGCTCTTCTATGGACTGCAGA -3` |
| UQCRC2 | forward:5`- ATTGATTCAGTGGCTAATGCT -3` |
|  | reverse: 5`- AAATTTCCACTTGCTGCCAT -3` |
| MT-CYB | forward:5`- GGGGCCACAGTAATTACAAA -3` |
|  | reverse: 5`- GGGGGTTGTTTGATCCCGTTT -3` |
| MTCO2 | forward:5`- CTGAACCTACGAGTACACCG -3` |
|  | reverse: 5`- TTAAGGCGACAGCGATTTCT -3` |
| ATP5A1 | forward:5`- TGTCCTCTATTCTTGAAGAGCGTA -3` |
|  | reverse: 5`- AATACTTAAGACACGCCCAGT -3` |
| MTATP6 | forward:5`- CGCCACCCTAGCAATATCAA -3` |
|  | reverse: 5`- TTAAGGCGACAGCGATTTCT -3` |
| POLRMT | forward:5`- GGACTCCAAGGTCAAGCAAATAGGAG -3` |
|  | reverse: 5`- AGGTCGAAGGCCCCTGGCTTG -3` |
| NRF1 | forward:5`- TTTGCTTCGGAAACTTCGAG -3` |
|  | reverse: 5`- ACATTCTCCAAAGGTGCTG -3` |
| TFB2M | forward:5`- TTGACTCCACTTGATGCGAGA -3` |
|  | reverse: 5`- CACAATCTTTGGAACGCTCT -3` |
| TFB1M | forward:5`- CTGCCTTTTAGTGTTTCAACTCC -3` |
|  | reverse: 5`- CTGCCATAAACAAAAGGTCCA -3` |
